# The Role of Simvastatin in the Therapeutic Approach of Rheumatoid Arthritis

**DOI:** 10.1155/2013/326258

**Published:** 2013-06-12

**Authors:** Lucia Cojocaru, Andrei Constantin Rusali, Cristina Şuţa, Anca Mihaela Rădulescu, Maria Şuţa, Elvira Craiu

**Affiliations:** ^1^Internal-Medicine Cardiology, Ovidius University of Constanta, Faculty of Medicine, Cardiology Clinic, Emergency Clinical County Hospital of Constanta, 900591 Constanta, Romania; ^2^Internal-Medicine Rheumatology, Ovidius University of Constanta, 900591 Constanta, Romania; ^3^Cardiology Clinic, Emergency Clinical County Hospital of Constanta, 900591 Constanta, Romania; ^4^Internal Medicine-Rheumatology, Ovidius University of Constanta, Faculty of Medicine, 2nd Medical Clinic, Emergency Clinical County Hospital of Constanta, 900591 Constanta, Romania

## Abstract

The pleiotropic effects of statins, especially the anti-inflammatory and immunomodulatory ones, indicate that their therapeutic potential might extend beyond cholesterol lowering and cardiovascular disease to other inflammatory disorders such as rheumatoid arthritis. Therefore, we undertook a prospective cohort study to evaluate the efficacy and safety of simvastatin used for inflammation control in patients with rheumatoid arthritis. One hundred patients with active rheumatoid arthritis divided into two equal groups (the study one who received 20 mg/day of simvastatin in addition to prior DMARDs and the control one) were followed up over six months during three study visits. The results of the study support the fact that simvastatin at a dose of 20 mg/day has a low anti-inflammatory effect in patients with rheumatoid arthritis with a good safety profile.

## 1. Introduction

Since their discovery in 1976, 3-hydroxy-3methylglutaryl-coenzyme A reductase inhibitors (or statins) have emerged as the leading therapeutic regimen for treating hypercholesterolemia modifying, an important cardiovascular risk factor with subsequent reduction of cardiovascular morbidity and mortality [1]. Numerous clinical studies have demonstrated the efficiency of statins in this context, in both primary [2–4] and secondary prevention strategies [5–17]. In parallel, it has become increasingly apparent that the beneficial effects of statins in cardiovascular pathology cannot be ascribed solely to their lipid-lowering properties, but also to another mode of action. These so-called “pleiotropic effects” which encompass modification of endothelial function, plaque stability and thrombus formation, and anti-inflammatory and immunomodulatory properties indicate that the therapeutic potential of statins might extend beyond cholesterol lowering and cardiovascular disease to other inflammatory disorders or conditions such as transplantation, multiple sclerosis, rheumatoid arthritis, systemic lupus erythematosus, and chronic kidney disease [18]. Extensive *in vitro* [19, 20] and *in vivo* [21–25] data sets support this statement.

Rheumatoid arthritis, the commonest of the inflammatory arthritides, is a chronic, systemic, inflammatory disorder which has as primary target the synovial tissue. The characteristic of rheumatoid arthritis is the persistent inflammation of the peripheral joints which leads to pain, stiffness, and swelling, with their gradual destruction, and, in time, it may lead to joint deformities and functional disability. Although the majority of physicians' efforts had been targeted at symptom control and reduction of joint damage, it has been known for more than 50 years that rheumatoid arthritis is associated with significantly increased mortality rates compared with the general population [26]. An important part that accounts for this excess mortality is an increase in cardiovascular deaths [27]. Their increased cardiovascular morbidity and mortality [28] are primarily due to accelerated atherosclerosis [29] which develops due to a complex interaction between traditional risk factors (dyslipidaemia, diabetes mellitus, hypertension, smoking, sedentary lifestyle, and obesity) and those related to the inflammatory disease [30, 31].

Despite advances in the treatment of rheumatoid arthritis, its mortality does not appear to have significantly changed over the last few decades, highlighting the need for closer attention to prevention and treatment of cardiovascular events in these patients [32]. Thus, optimal treatment of rheumatoid arthritis should reasonably deal with vascular risk modification in addition to the well-recognized objectives of treatment, namely, to suppress inflammation, improve function, prevent articular damage, and modify psychosocial implications of the disease [33]. The already known cardiovascular protective effect together with the new emerged anti-inflammatory one may render statins an attractive adjunct therapy in rheumatoid arthritis. Up to date, few clinical studies support this statement [34–39]; nevertheless, further clinical trials are required to verify whether more widespread use of statins should be recommended in patients with rheumatoid arthritis. Therefore, we undertook a cohort prospective study which had as primary objectives the reduction in disease activity (measured by the proportion of patients meeting EULAR response criteria and the change in the DAS28 ESR scale, a validated composite disease activity score that incorporates erythrocyte sedimentation rate, patient global assessment of disease activity, visual analogue scale for pain, and tender and swollen joint count based on the evaluation of 28 joints) and the evaluation of the frequency and severity of adverse events; among the secondary outcome measures there were change in composite indices of disease activity assessment (DAS28 CRP, SDAI, and CDAI), change in clinical variables of disease activity (duration of morning stiffness, tender joint count, swollen joint count, HAQ-DI, measure of pain using a visual analogue scale, and patient and evaluator global assessments of disease activity), change in acute phase reactants (C-reactive protein and erythrocyte sedimentation rate), change in rheumatoid factor and anticyclic citrullinated peptide antibodies levels, and change in lipid profile and incidence of cardiovascular events.

## 2. Material and Methods

**Figure 1:**
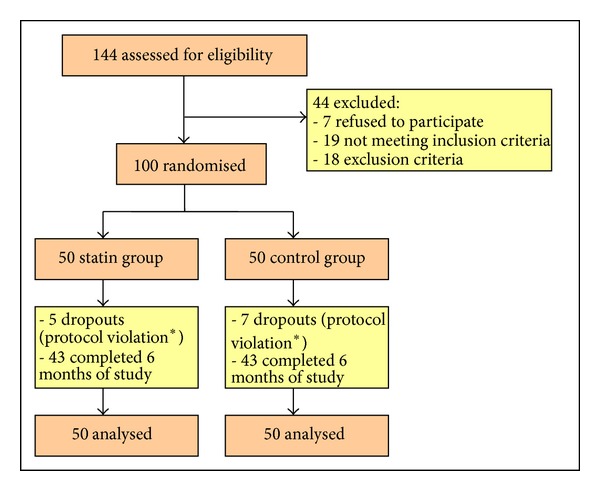
Study profile; *modification of DMARDs.

The clinical study was performed in Rheumatology Department of 3rd Medical Clinic of Emergency Clinical Hospital of Constanta from October 2008 to October 2009. From October 2008 to April 2009, 144 patients were screened (122 women and 22 men) (Figure 1). After giving the informed consent, we recruited 100 patients with ages between 18 and 80 years, meeting the 1987 American College of Rheumatology criteria, with active inflammatory disease (defined by disease activity score—DAS28—greater than 2.4) despite ongoing DMARD therapy, in adequate doses for at least 3 months for hydroxychloroquine, or 4 weeks for methotrexate, sulfasalazine, leflunomide, or etanercept. The exclusion criteria were diabetes, familial hypercholesterolaemia, use of oral prednisolone >10 mg/day (or doses ≤ 10 mg that were not stable for 4 weeks prior to screening visit), intra-articular cortisone injections within 4 weeks of study entry, statin therapy in the last 3 months prior to the study or with previous adverse reaction to statins, active or recent infection, myositis or elevation in creatine phosphokinase more than twice the upper limit of normal range, liver disease or abnormal liver function (transaminases > 2 times the upper limit of normal range), alcohol abuse, high serum creatinine level, chronic disorders other than rheumatoid arthritis affecting the joints, pregnancy or breastfeeding, and inclusion in other studies with investigational products. During the study, patients remained on all DMARDs in constant doses, nonsteroidal anti-inflammatory drugs, drugs for comorbid conditions, and oral prednisolone (in doses ≤ 10 mg/day) from study entry.

The 100 patients included in the study were divided into two equal groups (one group received simvastatin 20 mg/day in addition to prior DMARD therapy and the other, the control group, remained on the same DMARD therapy from the study entry) taking into account the indications of statin therapy [40]. The patients were followed for 6 months, during 3 study visits (at the inclusion, at 3 months, and at 6 months). At each visit, we performed anamnesis with visual analog pain scale, health assessment questionnaire disability index, patient's global assessment of disease activity, physical exam (measuring vital signs and clinical variables of disease activity), and evaluator global assessments of disease activity; we harvested blood samples for laboratory tests (hemogram, C-reactive protein, erythrocyte sedimentation rate, transaminases, creatine phosphokinase, and, only at inclusion and a 6-month visit: lipid profile, rheumatoid factor, anticyclic citrullinated peptide antibodies, and serum creatinine levels) and we calculated disease activity indices, SDAI, CDAI, and DAS28 (using ESR and CRP). During the study and in the statin group, there were 5 dropouts (two in the first 3 months and three in the last 3 months of study), and 95 patients completed the 6-month visit, and in the control group, there were 7 dropouts (one in the first 3 months and six in the last 3 months of study), and 93 completed the 6-month visit (Figure 1).

All data were processed statistically with the aid of SPSS v.17.0. The data were analyzed by the intention-to-treat approach. Patients who did not complete 6 months of study were categorised as nonresponders. For change in laboratory variables, we accounted for dropouts by carrying their baseline value forward to 6 months, no change was assumed.

## 3. Results and Discussions

Baseline characteristics of the study groups are shown in Table 1. There were no significant differences between the simvastatin and control groups except for: the lipid levels, proportion of hypertensive patients, proportion of patients with known cardiovascular disease, and the proportion of overweight patients (that were significantly higher in the simvastatin group as expected from the study design). The final results remained significant after the adjustment for these variables. Note that there were no significant differences between the two groups regarding the DMARDs use, thus removing an important element of confusion.

Table 2 documents outcomes after 3 and 6 months of study.

After 3 months of study, DAS28 ESR improved significantly (*P* = 0.009) in the statin group (−0.560; 95% CI = −0.849, −0.270; *P* < 0.001) when compared to the control one (0.076; 95% CI = −0.312, 0.465; *P* = 0.693; difference between groups = −0.636; 95% CI = −1.112, −0.160). But, in the following 3 months, DAS28 ESR recorded a significant increase in the statin group (0.24; 95% CI 0.012, 0.479; *P* = 0.039), while in the control group, it remained practically unchanged (−0.003; 95% CI −0.441, 0.434; *P* = 0.987), and so, the final benefic effect of simvastatin on this index was reduced. After 6 months of study the decreasing tendency of DAS28 ESR in the statin group (−0.313; 95% CI = −0.641, 0.013; *P* = 0.06) compared with the slight increasing tendency in the control group (0.073; 95% CI = −0.391, 0.537; *P* = 0.752) did not achieve statistical significance (mean difference between groups = 0.386; 95% CI = −0.170, 0.943; *P* = 0.171).

Regarding EULAR response criteria, after 3 months of study, moderate or good DAS28 responses were achieved in 16 of 50 (32%) patients allocated simvastatin compared with 7 of 50 (14%) patients in the control group (OR 2.89; 95% CI 1.06–7.82; *P* = 0.03). At the end of the study, there was no significant difference between the number of patients who achieved EULAR response criteria in the statin group—15 (30%), and those from the control group—11 (22%) (OR 1.51; 95% CI 0.61–3.74; *P* = 0.362). There were no significant differences between the two groups regarding the number of patients who completed the study (90% in the statin group versus 86% in the control one (*P* = 0.558)).

After 3 months of treatment, in the statin group, there was a significant improvement in DAS28 CRP (difference between groups = −0.574; 95% CI = −1.058, −0.091; *P* = 0.02), SDAI (difference between groups = −5.659; 95% CI = −9.307, −2.012; *P* = 0.003), CDAI (difference between groups = −5.379; 95% CI= −8.987, −1.772; *P* = 0.004), evaluator global assessment of disease activity (difference between groups = −10.560; 95% CI = −14.955, −6.166; *P* < 0.001), and tender joint count (difference between groups = −2.775; 95% CI = −5.750, 0.200; *P* = 0.03); other variables (swollen joint count, visual analogue scale for pain, patient global assessment of disease activity, and erythrocyte sedimentation rate) tended to decrease more in the simvastatin group compared to the control one, without reaching statistical significance.

After 6 months of treatment there was no significant difference between the disease activity variables in the two groups, except for evaluator global assessment of disease activity (difference between groups = −9.54; 95% CI = −15.913, −3.184; *P* = 0.007). The decrease of early morning stiffness, tender joint count, swollen joint count, HAQ-DI, VAS pain, DAS28 CRP, SDAI, and CDAI was more pronounced in the simvastatin group, and the levels of CRP and ESR increased less compared to those in the control group.

These results show that a small daily dose of simvastatin has some beneficial effects on clinical and biological parameters of disease activity in patients with active forms of rheumatoid arthritis despite DMARDs therapy, but without statistical significance. The anti-inflammatory effect of simvastatin was inferior to that of atorvastatin in TARA study [38], but we have to take into account the difference between the pharmacokinetic properties of these two statins (despite the fact that simvastatin is more lipophilic than atorvastatin, its oral absorption is inferior and has a grater variance when compared to that of atorvastatin—42.5% ±42.5 versus 57.5% ±17.5—and more importantly, the plasma half time of simvastatin is only 1.9 hr, whereas the plasma halftime of atorvastatin is 14 hr) and the lower dose used (20 mg of simvastatin versus 40 mg of atorvastatin). Another difference can emerge from the fact that in TARA study in the atorvastatin group, a significant higher number of patients were on methotrexate compared to the placebo lot (*P* < 0.07).

**Figure 2:**
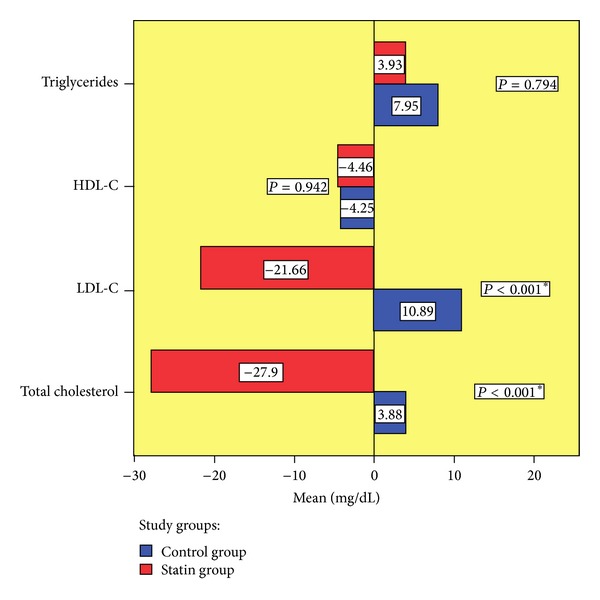
Evolution of lipid profile.

The evolution of lipid profiles after 6 months of study is presented in Figure 2. Total cholesterol and LDL-cholesterol were significantly reduced in simvastatin group compared to control group (total cholesterol − mean difference between groups = 31.77 mg/dL; 95% CI = 19.311, 44.243; *P* < 0.001; LDL-cholesterol − mean difference between groups = 32.54 mg/dL; 95% CI = 21.109, 43.972; *P* < 0.001). Regarding HDL cholesterol and triglycerides, there were no significant differences between the two groups. These results are inferior to the ones obtained in other clinical trials with simvastatin in patients without rheumatoid arthritis. For example, in 4S Study [11] a daily dose of 20 mg simvastatin (Zocor, Merck Sharp & Dohme) for 6 weeks significanty (*P* < 0.0001) decreased the level of total cholesterol (by 28%) and LDL cholesterol (by 38%) and increased the level of HDL cholesterol (by 8%). So we can conclude that statins maintain their hypocholesterolemic effect in context of high-grade inflammatory state like that present in rheumatoid arthritis, but their efficacy may be reduced when compared with that in other patients; so, there may be need for increased dose of statins for the treatment of hypercholesterolaemia in patients with rheumatoid arthritis.

During the study, simvastatin was well tolerated. Study dropouts were due to protocol violation, namely, the necessity of DMARDs modification and not because of simvastatin use. Adverse events were mild and arose with similar frequency in both groups (Table 3). In particular, no significant liver function or muscle abnormality was detected in those given simvastatin. Also, during the study there were no significant cardiovascular events.

## 4. Conclusions

Simvastatin at a dose of 20 mg/day has small anti-inflammatory effects in patients with rheumatoid arthritis. These effects do not have the magnitude to justify neither the use of statins instead of DMARDs therapy nor their routine use in all patients with rheumatoid arthritis. But thanks to good safety profile, easy administration, and the existence of a broad experience regarding their use in clinical practice, statins are particularly attractive therapeutic agents, so that even a modest efficacy in the treatment of rheumatoid arthritis in association with the reduction of cardiovascular risk can lead to a beneficial therapeutic ratio. This can make statins become particularly useful as adjuvant therapy associated with other conventional therapeutic methods used in rheumatoid arthritis, especially in patients with dyslipidaemia, where they should be the first choice of treatment.

## Figures and Tables

**Table 1:** Baseline characteristics.

Variable	Simvastatin group	Control group	*P*
Age (years)	61.15 (9.49)	56.48 (11.84)	0.053
Women	44 (88%)	42 (84%)	0.566
Rheumatoid factor positive	25 (50%)	27 (54%)	0.69
Anti-CCP antibody positive	25 (50%)	28 (56%)	0.55
Disease duration (years)	10.62 (9.37)	11.98 (11.86)	0.956
Methotrexate	32 (64%)	33 (66%)	0.835
Sulfasalazine	20 (40%)	19 (38%)	0.838
Hydroxychloroquine	10 (20%)	11 (22%)	0.807
Leflunomide	12 (24%)	11 (22%)	0.813
Etanercept	1 (2%)	1 (2%)	1
Oral prednisolone	16 (32%)	17 (34%)	0.832
Early morning stiffness (min)*	12.80 (0–60)	15.25 (0–60)	0.446
Tender joint count*	7.02 (0–24)	5.23 (0–25)	0.168
Swollen joint count*	1 (0–8)	0.6 (0–4)	0.684
VAS pain (mm)	59.76 (18.90)	50.50 (23.74)	0.062
Patient global assessment (mm)	58.29 (14.47)	54.75 (19.61)	0.206
Evaluator global assessment (mm)	51.46 (9.10)	47.50 (12.55)	0.066
HAQ-DI	1.53 (0.51)	1.36 (0.59)	0.166
Erythrocyte sedimentation rate (mm/h)	27.76 (15.81)	27.58 (18.76)	0.545
C-reactive protein (mg/L)*	4.66 (0.36–36.67)	4.71 (0.6–21.6)	0.932
DAS28 ESR	4.44 (0.98)	4.04 (1.00)	0.074
DAS28 CRP	3.72 (1.00)	3.39 (0.91)	0.122
SDAI	19.51 (9.03)	16.19 (8.02)	0.085
CDAI	19.05 (9.10)	15.73 (8.09)	0.087
Disease activity			
LDA	14 (28%)	19 (38%)	0.290
MDA	28 (56%)	25 (50%)	0.550
HDA	8 (16%)	6 (12%)	0.566
Total cholesterol (mg/dL)	240.34 (40.33)	197.15 (30.13)	<0.001
LDL cholesterol (mg/dL)	155.07 (32.29)	113.66 (25.30)	<0.001
HDL cholesterol (mg/dL)	62.33 (15.88)	63.53 (19.06)	0.758
Triglyceride (mg/dL)	133.58 (49.22)	108.65 (48.29)	0.024
Total creatine phosphokinase (UI/L)	73.12 (33.44)	78 (51.74)	0.788
Alanine transaminase	17.65 (6.82)	22.74 (12.39)	0.055
Aspartate aminotransferase	19.53 (5.45)	21 (7.19)	0.576
Hypertension	42 (84%)	27 (54%)	0.001
Smokers	10 (20%)	9 (18%)	0.8
Ex-smokers	2 (4%)	3 (6%)	0.648
Overweight	26 (52%)	16 (32%)	0.044
Obesity	15 (30%)	13 (26%)	0.658
Previous stroke	2 (4%)	1 (2%)	0.56
Coronary heart disease	10 (20%)	0 (0%)	0.001

Between the breaks there are standard deviations or percents unless otherwise indicated; *minimal and maximal values of the variable; LDA: low disease activity; MDA: moderate disease activity; HDA: high disease activity.

**Table 2:** Differences after 3 and 6 months of treatment.

Variable	Follow-up visit	Simvastatin group	Control group	P
Early morning stiffness (min)	3 month	0.244 (−5.346; 5.834)	−1.125 (−9.962; 7.712)	0.63
	6 month	−3.536 (−7.701; 0.628)	−2.25 (−10.804; 6.304)	0.996
Tender joint count	3 month	−3 (−4.894; − 1.106)	−0.225 (−2.591; 2.141)	**0.03**
	6 month	−1.414 (−3.513; 0.684)	1.650 (−1.009; 4.309)	0.243
Swollen joint count	3 month	−0.561 (−1.143; 0.021)	0.275 (−0.431; 0.981)	0.068
	6 month	−0.341 (−0.808; 0.125)	0.050 (−0.425; 0.525)	0.288
VAS pain (mm)	3 month	−1.219 (−8.762; 6.323)	3.75 (−4.561; 12.061)	0.888
	6 month	−1.219 (−7.421; 4.982)	4.500 (−4.745; 13.745)	0.481
Patient global assessment (mm)	3 month	−4.39 (−10.255; 1.475)	0.25 (−6.084; 6.584)	0.273
	6 month	−2.926 (−8.714; 2.861)	−5.00 (−13.900; 3.900)	0.196
Evaluator global assessment (mm)	3 month	−7.561 (−10.620; −4.502)	3 (−0.255; 6.255)	**<0.001**
	6 month	−8.049 (−12.412; −3.685)	1.500 (−3.279; 6.279)	**0.007**
HAQ-DI	3 month	−0.064 (−0.172; 0.043)	−0.122 (−0.288; 0.043)	0.353
	6 month	−0.153 (−0.283; −0.023)	−0.019 (−0.194; 0.154)	0.149
Erythrocyte sedimentation rate (mm/h)	3 month	−0.926 (−4.653; 2.799)	2.125 (−2.712; 6.962)	0.203
	6 month	2.829 (−1.046; 6.705)	3.800 (−1.018; 8.618)	0.751
C-reactive protein (mg/L)	3 month	0.961 (−0.178; −2.101)	3.767 (1.506; −6.027)	0.055
	6 month	1.676 (−0.833; 4.186)	2.57 (0.641; 4.499)	0.687
DAS28 ESR	3 month	−0.56 (−0.849; −0.270)	0.076 (−0.312; 0.465)	**0.009**
	6 month	−0.313 (−0.641; 0.013)	0.073 (−0.391; 0.537)	0.171
DAS28 CRP	3 month	−0.484 (−0.771; −0.196)	0.090 (−0.309; 0.491)	**0.02**
	6 month	−0.295 (−0.637; 0.045)	0.098 (−0.334; 0.531)	0.151
SDAI	3 month	−4.708 (−6.889; −2.527)	0.951 (−2.061; 3.963)	**0.003**
	6 month	−2.466 (−5.257; 0.324)	1.807 (−1.939; 5.553)	0.067
CDAI	3 month	−4.805 (−6.972; −2.637)	0.575 (−2.397; 3.547)	**0.004**
	6 month	−2.634 (−5.355; 0.086)	1.55 (−2.139; 5.239)	0.068
EULAR response	3 month	16 (32%)	7 (14%)	**0.033**
	6 month	15 (30%)	11 (22%)	0.364
Attendance to visit	3 month	48 (96%)	49 (98%)	0.560
	6 month	45 (90%)	43 (86%)	0.540

Between the breaks, there are 95% confidence interval or percents.

**Table 3:** Adverse events.

Adverse event	Simvastatin group	Control group	*P*
Elevated aspartate aminotransferase	1 (2%)	2 (4%)	0.558
Elevated alanine aminotransferase	1 (2%)	5 (10%)	0.092
Elevated creatine phosphokinase	1 (2%)	2 (4%)	0.558
Myalgia	2 (4%)	3 (6%)	0.646
Muscle weakness	1 (2%)	2 (4%)	0.558
Abdominal pain	4 (8%)	6 (12%)	0.505
Nausea	6 (12%)	4 (8%)	0.505
Constipation	0 (0%)	1 (2%)	0.315
Diarrhea	0 (0%)	1 (2%)	0.315
Flatulence	1 (2%)	2 (4%)	0.558
Asthenia	2 (4%)	4 (8%)	0.400
Dizziness	1 (2%)	3 (6%)	0.307
Headache	2 (4%)	4 (8%)	0.400
Allergy	0 (0%)	1 (2%)	0.315

## Abbreviations

CDAI: Clinical disease activity index
DAS28 CRP: Disease activity score based on the count of 28 joint and C-reactive protein
DAS28 ESR: Disease activity score based on the count of 28 joint and erythrocyte sedimentation rate
DMARDs: Disease-modifying antirheumatic drugs
EULAR: European league against rheumatism
HAQ-DI: Health assessmentquestionnaire-disability Index
HMG-CoA: 3-Hydroxy-3methylglutaryl-coenzyme A
SDAI: Simplified disease activity index.
